# Effects of Augmented Reality Motor Training on Gait and Balance in Children with Cerebral Palsy: Randomized Controlled Trial

**DOI:** 10.3390/children13091164

**Published:** 2026-08-29

**Authors:** Rudolf Psotta, Monika Šorfová, Josef Kraus, Marek Bureš, Natálie Cibulková, David Prycl

**Affiliations:** 1 Department of Wellness, College of Physical Education and Sport PALESTRA, 197 00 Prague, Czech Republic; 2 Department of Biomedicine, College of Physical Education and Sport PALESTRA, 197 00 Prague, Czech Republic; sorfova@palestra.cz (M.Š.); articlecze@gmail.com (N.C.); 3 Department of Paediatric Neurology, 2nd Faculty of Medicine, University Hospital Prague—Motol, Charles University, 150 06 Prague, Czech Republic; josef.kraus@lfmotol.cuni.cz; 4 XR Institute, Ltd., 301 00 Plzeň, Czech Republic; bures@xr-institute.cz

**Keywords:** motor training, augmented reality technology, cerebral palsy, gait, balance

## Abstract

**Highlights:**

**What are the main findings?**

- Adding four weeks of augmented reality motor training (ARMT) to conventional rehabilitation was associated with selective between-group differences in gait parameters, including bilateral stride cycle duration, right double-support variability, and propulsion index.
- However, no additional effects of ARMT were observed on overall balance, walking speed, stride length, gait quality, or gait symmetry.

**What are the implications of the main findings?**

- The short-term ARMT did not demonstrate comprehensive additional benefits over conventional rehabilitation in children with spastic cerebral palsy.
- The observed effects of ARMT on specific gait parameters should be considered exploratory results that require next confirmation in larger trials with pre-specified primary outcomes.

**Abstract:**

**Background**: The current evidence regarding the impact of augmented reality (AR) rehabilitation on motor function in children with cerebral palsy (CP) remains limited. **Objectives:** This study aimed to evaluate whether integrating AR motor training (ARMT) into conventional rehabilitation enhances gait and balance outcomes compared to conventional rehabilitation alone. **Methods:** Forty children aged 7 to 12 years with unilateral or bilateral spastic CP were randomly assigned to receive either ARMT integrated into a 4-week conventional rehabilitation program (n = 20) or conventional rehabilitation alone (n = 20). The ARMT replaced 20–30 min of standard motor therapy five days per week. Gait and balance were assessed pre- and post-intervention using an instrumented 10 m walk test with the G-Walk sensor, the MABC-2 one-leg balance task, and the Pediatric Balance Scale. Due to unavailability of data from five control-group participants, analyses were conducted on 35 children using available-case mixed-model approaches. **Results**: Nominally significant between-group differences in pre- to post-intervention changes were observed for left stride cycle duration (mean difference: −0.083 s; 95% confidence interval [CI]: [−0.166, −0.0002]; *p* = 0.049), right stride cycle duration (−0.095 s; 95% CI: [−0.185, −0.006]; *p* = 0.038), the coefficient of variation of the first double-support phase on the right side (−8.91 percentage points; 95% CI: [−17.39, −0.44]; *p* = 0.040), and the left propulsion index (2.42 m/s^2^; 95% CI: [0.39, 4.45]; *p* = 0.021). No statistically significant differences were detected between groups for balance measures, gait speed, stride length, gait quality, or symmetry. **Conclusions:** Incorporation of four weeks of ARMT into conventional rehabilitation did not yield substantial additional benefits for gait or balance in this cohort. The observed effects on gait timing, variability, and propulsion are exploratory and warrant validation in larger, adequately powered studies with pre-specified primary outcomes.

## 1. Introduction

Cerebral palsy (CP) is an umbrella term for disorders caused by brain damage or malformation occurring during the prenatal, perinatal, or early postnatal periods. It is characterized primarily by motor and postural impairments that limit activities of daily living. The fundamental pathophysiological classification of CP divides it into spastic, dyskinetic, and ataxic forms, with the highest incidence observed in spastic CP (75–85%).

Walking, as a fundamental motor function, presents significant challenges in children with spastic diparesis and hemiparesis. Children with bilateral spastic CP often exhibit markedly reduced gait speed, shorter step length, excessive co-contraction, and sagittal-plane deviations such as equinus, crouch gait, or jump gait [1,2]. These gait patterns are frequently accompanied by compensatory pelvic and trunk movements that increase energy expenditure.

In hemiparetic CP, asymmetrical involvement of the lower limbs results in different kinematic strategies between the affected and unaffected sides. Sangeux and Armand [2] described characteristic deviations, including reduced dorsiflexion and knee extension on the paretic side, limb circumduction during swing, and pelvic retraction. These adaptations aim to maintain foot clearance and forward progression but often reduce gait efficiency and symmetry.

Abnormal coordination of the pelvis and trunk is a hallmark of pathological gait in both diparetic and hemiparetic CP [3,4]. During gait in children with unilateral or bilateral spastic CP, abnormalities of the pelvis and trunk during the step phase can include pelvic tilt (double bump), pelvic obliquity (up or down), reversed pelvic rotation profiles, sustained pelvic protraction or retraction, trunk tilt (double bump), sustained trunk obliquity, and excessive trunk obliquity consistent with a Trendelenburg pattern [2]. These findings underscore the importance of pelvic and trunk dynamics in gait efficiency and highlight their relevance as primary targets in rehabilitation.

A key goal of rehabilitation is to improve mobility and ambulatory function. Contemporary approaches have shifted from solely impairment-based interventions—such as isolated strengthening exercises or joint range-of-motion enhancement—to strategies that directly target functional components of activity and participation. The integration of virtual reality (VR) environments can further enhance patient engagement, particularly in pediatric rehabilitation [5]. Therapeutic interventions in this context include home-based exercise programs delivered via digital platforms, sit-to-stand training protocols, and task-oriented functional exercises [6].

Rehabilitation programs aimed at improving functional walking in children with CP typically focus on intensive, task-specific gait training. A review and meta-analysis by Booth et al. [5] found that such interventions consistently improved walking speed, endurance, and gross motor function compared to standard physiotherapy. These interventions are usually conducted for about 30 min, 2–3 times per week, over a 12-week period. The review emphasized that functional gait training is most effective when repetitive, contextually relevant, and incorporating real-world walking tasks. Paul et al. [7] confirmed significant improvements in cadence, stride length, and velocity across various intervention modalities, reinforcing the importance of task-specific practice for enhancing functional mobility.

While gross motor improvements are well documented, fewer studies have directly examined how rehabilitation influences pelvic kinematics. Existing evidence suggests that interventions targeting lower-limb function often lead to secondary improvements in pelvic control. Kiernan et al. [4] noted the interdependence of trunk and pelvic motions with lower-limb mechanics, indicating that gait training improving limb alignment may reduce excessive lateral trunk displacement. Swinnen et al. [3] proposed that enhancing thorax and pelvis coordination could improve dynamic stability and reduce energy expenditure. Booth et al. [5] further suggested that functional gait training—including overground walking and postural transitions—may inherently promote proximal control.

Traditional therapy, although effective, can be monotonous and physically demanding. Virtual reality (VR) and augmented reality (AR) technologies offer immersive environments that can sustain motivation and facilitate motor learning. Current systems utilize head-mounted devices (HMDs) to project virtual scenes into the real world (AR) or create fully immersive digital environments (VR). Unlike earlier, less advanced devices, modern immersive technologies provide first-person perspective visualization, minimizing visuospatial transformations from performed movements to their virtual representations [8]. This can reduce cognitive load and accelerate motor learning [8].

Studies employing VR have demonstrated significant improvements in postural control, balance, walking performance, and gross motor function compared to standard therapy [9,10,11]. The mechanisms behind these benefits include multisensory feedback, enriched environments, and real-time visual reinforcement of correct motor patterns, all of which enhance sensorimotor integration and cortical reorganization.

The primary difference between VR and AR lies in their digital representation: VR provides a fully digital, immersive 3D simulation of the real world, while AR overlays digital information onto the user’s real environment in real time [12]. VR isolates the user within a digital environment, whereas AR combines digital elements with the physical world [12,13]. The potential advantage of AR is that it maintains the user in a real-world context, which may be particularly beneficial for individuals with motor impairments. AR allows movements to be performed within more ecologically valid settings, potentially reducing the risk of motion sickness and facilitating the transfer of practiced skills to daily activities [14,15].

Previous research using AR for balance or gait training has confirmed its safety, applicability, feasibility, and high adherence among older adults [16,17,18] and children with CP [19,20]. Only one study suggested that AR could improve postural stability in older adults [18].

There remains a gap in knowledge regarding the effects of AR-based motor training on the improvement of motor functions in children with cerebral palsy. Specifically, no studies have examined the impact of exercises performed within an AR environment as part of motor rehabilitation for children with motor impairments, particularly those with CP. Therefore, our study aimed to investigate the effects of augmented reality (AR) on gait and balance in children with CP. Based on the potential benefits of virtual, immersive technologies and the unique characteristics of AR, we hypothesized that incorporating AR-based motor exercises into a rehabilitation program could be more effective in improving balance and gait than conventional rehabilitation alone in children with CP. The goal of the study was to assess whether a short-term, 4-week AR motor training (ARMT) program could be effective and to determine the extent of its benefits.

## 2. Materials and Methods

### 2.1. Participants

The recruitment of children took place over a period of ten months at two pediatric rehabilitation centers. A total of 69 children who met the primary inclusion criteria—namely, age between 7 and 12 years and a diagnosis of spastic cerebral palsy with hemiplegia or diplegia—were assessed for additional eligibility criteria as follows: (1) Gross Motor Function Classification System (GMFCS) levels I–II; (2) spasticity graded at 2 or less according to the Modified Ashworth Scale (MAS); (3) ability to walk at least 10 m; (4) absence of neurological conditions other than cerebral palsy; (5) cognitive ability to communicate, at least with simple language; and (6) ability to follow instructions. Exclusion criteria included: (1) receipt of anti-spastic medication injections within the past three months; (2) orthopedic surgery within the previous year; (3) visual impairments that would prevent participation in interventions using a virtual headset and/or testing. Twenty-nine children were excluded for either not meeting the inclusion criteria or meeting the exclusion criteria.

Forty children who satisfied all inclusion and none of the exclusion criteria were randomly assigned to either the augmented reality motor training (ARMT) group or the control group, with an allocation ratio of 1:1. No formal a priori power calculation was performed. The target sample size of 40 participants was selected based on sample sizes used in previous studies examining VR-based rehabilitation in children with cerebral palsy [5,9,11,21]. The allocation sequence was generated using random-number generator by an investigator who was not involved in participant recruitment or assessment. Group assignments were concealed from the recruiting personnel until each participant had been enrolled and eligibility confirmed. No stratification was applied based on age, sex, unilateral or bilateral spastic cerebral palsy, or GMFCS level. Blinding was maintained such that therapists, parents, and children were unaware of group assignments prior to allocation.

During the planned four-week rehabilitation period, five children allocated to the control group discontinued participation due to reasons including: (1) common infectious respiratory disease (three children), (2) a leg fracture (one child), and (3) withdrawal by a parent prior to post-intervention assessment for family reasons (one child). Baseline and post-intervention outcome data were unavailable for these five participants. Consequently, the analyses included only those participants with available pre- and post-intervention measurements, comprising 20 children in the ARMT group and 15 children in the control group. Table 1 presents the basic clinical characteristics of the groups.

This study was approved by the Institutional Ethical Committee (approval code VSP/2151/2024) and by the Technology Agency of the Czech Republic (grant number TQ01000031). Informed consent was obtained from the legal guardians of all participating children following detailed information regarding walking and balance assessments and the ARMT intervention. The study was registered with ClinicalTrials.gov under the identifier NCT 07664878.

### 2.2. Apparatus

**Figure 1:**
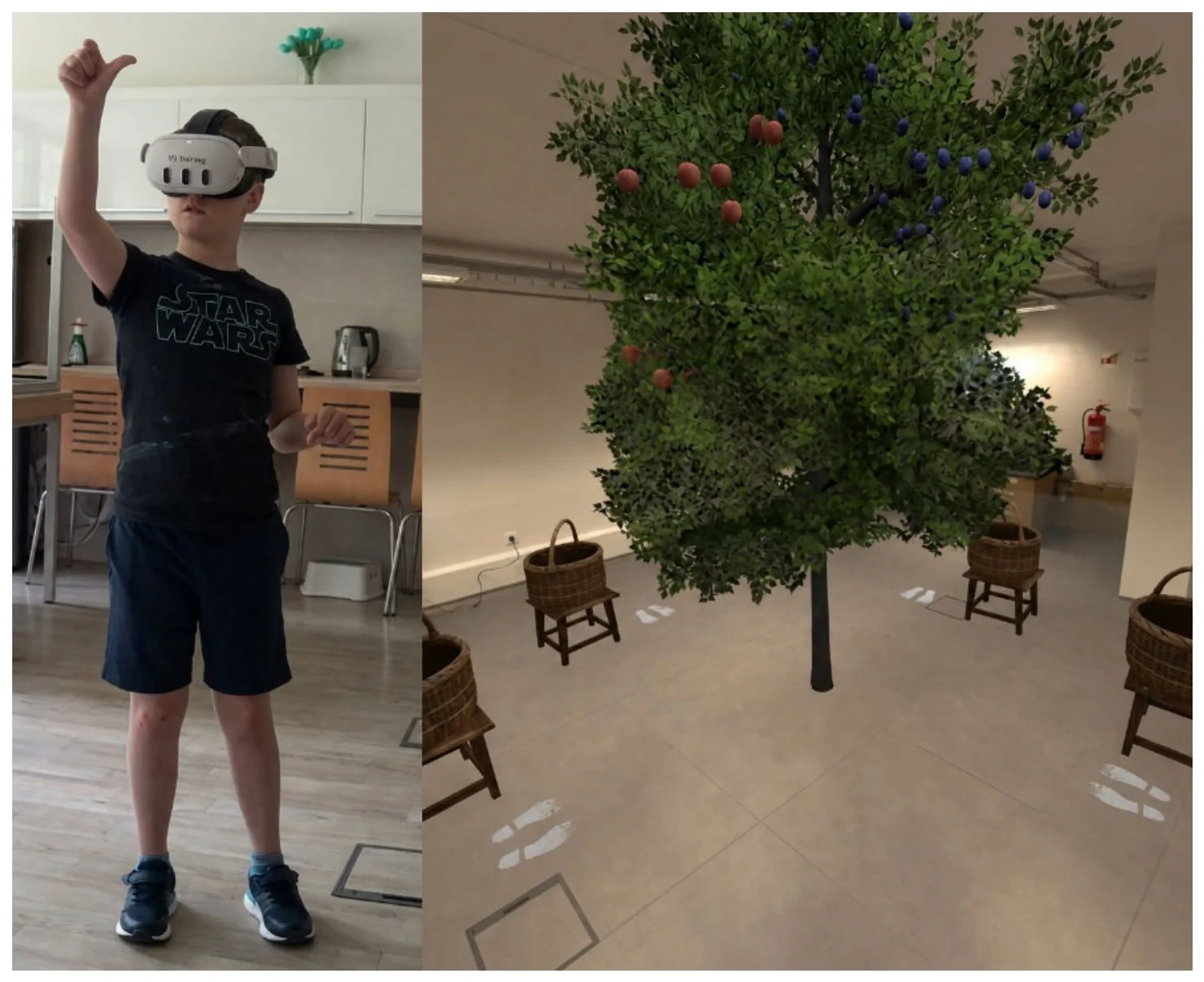
Movement task “Picking fruit” to stimulate balance. The child was collecting fruit with one hand from each of the four sides of the tree while two-leg standing, standing with feet together, heel-to-toe standing, and one-leg standing.

The software applications (apps) for augmented reality (AR)-based motor exercises included four exercises designed for this study to stimulate walking ability—namely, “Walking through a fallen hollow tree trunk,” “Crossing a brook,” “Walking through a labyrinth,” and “Walking through a narrow gorge”—as well as three exercises aimed at enhancing balance: “Picking fruit from a tree while standing in different positions,” “Turning a body after a balloon,” and “Bimanual tracking of balloons with body turning” (Figure 1). Each exercise was adaptable to three levels of difficulty, resulting in a total of 21 exercise variations.

To practice motor tasks in augmented reality (AR), the participant wore a Meta Quest 3 VR/AR headset (Meta Platforms, Menlo Park, CA, USA), on which the aforementioned software (SW) motor exercise applications were launched. The Meta Quest 3 is an all-in-one device equipped with four infrared (IR) cameras (400 × 400 pixels) for tracking controllers, hands, and spatial positioning, as well as two 4-megapixel RGB cameras for VR/AR functionalities. Each eye’s display resolution is 2064 × 2208 pixels, with a refresh rate of 90 Hz. The inter-pupillary distance of the lenses is adjustable within a range of 53 to 75 mm. The headset has a horizontal field of view of 110° and a vertical field of view of 96°, allowing the user to perceive the environment in a manner similar to natural vision. The device weighs 515 g.

Each application provides real-time dynamic visuospatial feedback and auditory cues, such as signals when the virtual environment—e.g., the interior of a hollow tree trunk that the participant is intended to walk through—is touched. Additionally, the applications deliver brief verbal instructions at the start of each exercise and offer verbal encouragement to promote external focus of attention, such as emphasizing the intended movement effect, outcome, implement, or goal (see [22]). This approach aims to enhance intrinsic motivation [23] and facilitate the acquisition of motor skills [22,23]. The physiotherapist utilized a tablet running the control software platform to select specific exercises, adjust their difficulty levels (ranging from 1 to 3), and set durations (from 1 to 5 min). From the tablet interface, the physiotherapist could monitor in real time what the participant was viewing through the headset.

### 2.3. Augmented Reality Motor Training (ARMT)

Both groups participated in a four-week conventional therapeutic program designed to stimulate fundamental motor and neuromuscular functions. This standardized program was employed across both pediatric rehabilitation institutions. It comprised traditional occupational and motor therapy conducted four times weekly, mechano-therapy three times weekly, manual massage twice weekly, swimming exercises twice weekly, whirlpool baths (hydro-massage) three times weekly, and pearl baths twice weekly.

For the ARMT group, additional focus on walking and balance/postural stability functions was integrated into the conventional rehabilitation program, while maintaining the total duration of the four-week therapeutic regimen consistent with that of the control group. The rehabilitation methods utilized in the conventional program were identical for both groups. The primary difference was that conventional motor therapy was reduced by 20–30 min per day in the ARMT group, with this time replaced by the ARMT intervention (active-treatment substitution). The ARMT program was developed and implemented by the authors in collaboration with medical and physiotherapy authorities from both rehabilitation institutions.

The ARMT was administered over five sessions per week, from Monday to Friday. Each session lasted between 20 and 30 min, during which the child performed 2–3 exercises within the augmented reality environment, involving varying repetitions. The entire ARMT comprised seven exercises, each with three levels of difficulty, resulting in a total of 21 specific movement tasks. The difficulty level of these tasks was progressively increased and adapted throughout the four-week training period in accordance with the child’s current abilities. Selection of the more challenging modification for each motor task was based on the following progression criteria: (i) observable improvements in movement quality as assessed by the physiotherapist, and (ii) quantitative indicators of movement performance in the specific task, such as decreased completion time, reduced variability in task duration (coefficient of variation, CV%), and the number of successful attempts within a designated period. These quantitative parameters were measured and recorded using the software applications associated with the ARMT. During each session, the physiotherapist provided verbal feedback to facilitate movement corrections. If necessary, short rest intervals were incorporated between tasks to optimize performance and prevent fatigue.

### 2.4. Assessment of Potential Effects

To evaluate the gait cycle, participants performed the 10 m walk test while wearing the G-Walk sensor device (BTS G-Walk, BTS Bioengineering, Milano, Italy). The G-Walk is equipped with a tri-axial accelerometer (16-bit resolution per axis, with multiple sensitivity settings), a tri-axial magnetometer (13-bit resolution, ±1200 μT), and a tri-axial gyroscope (16-bit resolution per axis, with sensitivity options of ±250, ±500, ±1000, and ±2000°/s). The device was affixed to an elastic belt and positioned below the line connecting the two dimples of Venus—corresponding to the lumbosacral junction (S1–S2 vertebrae), as specified in the manual [24]. Subsequently, the child was instructed to walk at a comfortable, self-selected pace along a 10 m track, with the boundaries marked by the yellow tape. A therapist accompanied the child as needed to ensure safety and maintain consistent walking speed. Each child completed two trials of the 10 m walk, with data from the second trial analyzed. If any unexpected movements—such as sneezing, coughing, stumbling, or near-stopping—were observed during a trial, it was repeated.

The G-Walk data were processed using G-Studio software (Version 3.2.25.0, BTS G-Walk, BTS Bioengineering, Italy), which provided spatiotemporal parameters of the stride cycle and pelvic movements across three planes: sagittal (tilt), coronal (obliquity), and transverse (rotation). The parameters included cadence (steps per minute), walking velocity (m/s), step length as a percentage of height, step duration (seconds), durations of stance and swing phases (both expressed as a percentage of the gait cycle), duration of double support (% cycle), propulsion index, walk-quality index, symmetry index, and pelvic tilt, obliquity, and rotation symmetry indices. All data were collected at a sampling frequency of 100 Hz.

Balance was assessed using the Pediatric Balance Scale (PBS) [25]. An experienced physiotherapist administered 14 balance tasks from the PBS, observing each child’s performance and rating it on a five-point scale according to the manual. Additionally, balance was evaluated through the “Balance on a Board” task from the Movement Assessment Battery for Children—Second Edition (MABC-2) [26]. This task required the child to stand on one leg on a plastic board (length: 31.5 cm; width: 10.0 cm; narrower side: 3.5 cm) placed flat on the floor. The objective was to maintain the position for as long as possible, up to a maximum of 30 s. The task was conducted in accordance with the MABC-2 manual [26], and the total time achieved across two trials was used for analysis.

### 2.5. Data Analysis

Longitudinal changes in the outcome measures were analyzed using outcome-specific mixed-effects models. Each model included fixed effects of time (pre- vs. post-intervention), group (ARMT vs. control), and their interaction, together with a participant-specific random intercept. The group-by-time interaction represented the estimated intervention effect. Model specification was selected separately for each outcome according to its measurement scale, bounds, and empirical distribution. Gaussian linear mixed models were used for approximately symmetric outcomes, including cadence, stride length, selected gait-phase measures, and pelvic tilt symmetry. Positively skewed outcomes, including walking speed, stride-cycle duration, most measures of gait variability and propulsion, and right- and left-leg MABC-2 balance durations, were analyzed using log-normal mixed models. Outcomes with a fixed optimal maximum were analyzed as deficits from that maximum: 100−y for gait-quality and symmetry indices and 56−y for the Pediatric Balance Scale. The percentage-based deficits were analyzed using log-normal models, whereas the Pediatric Balance Scale deficit was modelled using a Poisson mixed model. This outcome-specific approach made model-based parametric inference feasible in the limited sample without imposing a single distribution on heterogeneous outcome measures. After back-transformation, results from all models were presented consistently on their original measurement scales as estimated pre–post changes and between-group differences in change, with 95% confidence intervals and two-sided *p*-values.

The analyses were conducted using an available-case approach rather than an intention-to-treat approach. Because baseline and follow-up outcome data were unavailable for the five participants who discontinued from the control group, the analyses were restricted to participants with the measurements required for the respective model. No missing outcomes were imputed.

## 3. Results

Outcome analyses included 35 participants with available pretest and posttest data: 20 participants in the ARMT group and 15 participants in the control group.

### 3.1. Descriptive Results

Raw participant-level distributions of gait and balance outcomes at pretest and posttest are presented in Table 2. Values are summarized using the first quartile, median, and third quartile separately for the ARMT and control groups.

### 3.2. Model-Based Treatment Effects

Treatment effects were evaluated as differences in pretest-to-posttest changes between the ARMT and control groups. Within-group changes are reported in Table 3 to facilitate interpretation, but statistical inference regarding the effect of ARMT was based on the between-group difference in changes.

Four between-group contrasts reached the nominal two-sided significance threshold of α = 0.05. Compared with the control group, the ARMT group showed a greater reduction in stride cycle duration for both the left lower limb, with a difference in changes of −0.083 s (95% CI [−0.166, −0.0002], *p* = 0.049), and the right lower limb, with a difference of −0.095 s (95% CI [−0.185, −0.006], *p* = 0.038). A greater reduction in the coefficient of variation of the first double-support phase of the right lower limb was also observed in the ARMT group, with a difference of −8.91 percentage points (95% CI [−17.39, −0.44], *p* = 0.040). In addition, the left-limb propulsion index changed more favorably in the ARMT group, with a between-group difference of 2.42 m/s^2^ (95% CI [0.39, 4.45], *p* = 0.021).

Several additional effects were suggestive but did not reach the α = 0.05 threshold. These included one-leg balance duration for the right lower limb measured using the MABC-2 task, with a difference in changes of 4.96 s (95% CI [−0.28, 10.21], *p* = 0.063); the right-limb propulsion index, with a difference of 1.38 m/s^2^ (95% CI [−0.14, 2.91], *p* = 0.074); and the duration of the left single-support phase, with a difference of −2.37 percentage points of the stride cycle (95% CI [−5.01, 0.27], *p* = 0.077). Suggestive differences were also observed for left-leg MABC-2 balance duration (6.89 s, 95% CI [−1.06, 14.83], *p* = 0.088), the coefficient of variation of left stride cycle duration (−3.28 percentage points, 95% CI [−7.18, 0.62], *p* = 0.096), and the coefficient of variation of the left stance phase (−2.49 percentage points, 95% CI [−5.45, 0.47], *p* = 0.097).

No evidence of a between-group difference was found for cadence, walking speed, stride length, the total Pediatric Balance Scale score, walk-quality indices, symmetry indices, or the remaining gait-phase and variability outcomes (all *p* ≥ 0.10).

Because multiple gait and balance outcomes were evaluated and no adjustment for multiplicity was applied, the reported *p*-values are nominal and the findings should be interpreted as exploratory.

## 4. Discussion

The primary objective of this randomized controlled trial was to assess whether a brief, four-week augmented reality motor training (ARMT), incorporated into a standard rehabilitation framework, could yield improvements in walking ability, its consistency, and postural stability in children with CP. At baseline, both groups were well matched regarding basic anthropometric and clinical characteristics, including the degree of spasticity across the ankle, knee, elbow, and wrist joints. Additionally, prior to the commencement of the rehabilitation program, no significant differences were observed between the ARMT and control groups concerning gait pattern parameters and balance.

In the literature, evidence indicates that walking speed and cadence in children with CP are markedly reduced. For instance, the study by Kim and Son [27] reported that children with CP exhibit walking speed and cadence at approximately 60% and 77%, respectively, of those observed in typically developing children. Following a 4-week rehabilitation program incorporating motor exercises within an AR environment, the ARMT group demonstrated significant reductions in stride cycle durations for both the left and right sides, as well as a decrease in the coefficient of variation (CV%) of the first double-support duration on the right side and a significant increase in the propulsion index during left-side gait cycles, compared to the control group that received conventional rehabilitation. Generally, a reduction in stride cycle duration may reflect an increase in cadence or walking speed. In individuals with CP, such a reduction could be driven by a compensatory increase in cadence to maintain functional walking speed despite restricted stride length [28]. However, in the ARMT group, the reduction in stride cycle duration was associated not only with increased cadence but also with longer stride lengths and higher walking speeds, although these changes did not reach statistical significance at the α = 0.05 level. These outcomes were not observed in the control group. Additionally, 35% of children in the ARMT group achieved normalization of stride length, compared to only 7% in the control group. These findings suggest that the observed reduction in stride cycle duration, coupled with increased stride length, may contribute to an overall increase in walking speed.

The propulsion index, measured in meters per second squared (m·s^−2^) using the G-Walk Bioengineering sensor, serves as an indicator of a patient’s ability to fully accept body weight on a lower limb following deceleration and to propel the center of mass forward during the push-off phase [24]. This functional capacity reflects the generation of propulsive forces during the stance phase’s single-support period [28,29]. A higher propulsion index indicates greater progression capacity during this phase [24]. Our study observed a significant increase in the propulsion index for left-side gait cycles and a marginally significant increase (α = 0.074) for right-side gait cycles within the ARMT group. The biomechanical transfer of ground reaction forces from the stance leg to the contralateral limb is a key determinant of walking ability [30]. These findings may suggest improved force transfer during the initial double-support phase, facilitating push-off and swing, thereby contributing to increased walking speed. The significant enhancement in force transfer from the stance phase of the left leg to the opposite side may also be supported by the observed lower variability in the stance duration of the left leg.

Furthermore, the study identified a reduction in variability of the first double-support phase duration for the right leg in the ARMT group compared to controls. The percentage of double support is often used as a proxy for dynamic gait stability in individuals with neurological impairments [31]. The first double-support phase encompasses initial double limb support and loading response, beginning at heel strike and ending when the opposite foot lifts off [32]. Notably, the loading response and subsequent mid-stance are key intervals during which propulsion forces are generated from the stance leg to the contralateral limb. Although no significant changes were observed in the propulsion index of the right leg, the decreased variability in the duration of the first double-support phase may support improved stabilization of the body during force transfer from the right to the left side.

The one-legged stance performed at the beginning of rehabilitation revealed significant postural stability abnormalities in children with CP. Specifically, 95% and 85% of children in the ARMT group achieved stance durations corresponding to the ≤5th percentile of normative data for Czech children of similar age [33] for the left and right legs, respectively. In the control group, these figures were 86.7% and 73.3%. While some studies have reported beneficial effects of VR therapy on balance in children with spastic CP [11,21], our study did not demonstrate a statistically significant improvement in balance abilities following ARMT. Nonetheless, a marginal improvement, indicated by a slight prolongation of one-leg stance duration, was observed in both legs within the ARMT group.

Although no significant between-group effect was observed for the Pediatric Balance Scale (PBS) score, confidence intervals suggested an increase in this score among most children in the ARMT group, whereas a decrease was observed in a substantial portion of children in the control group (see Table 3). Additionally, a significantly higher percentage of children in the ARMT group demonstrated improvement in the single-leg and tandem stance tasks of the PBS compared to the control group (45.5% vs. 20.0% and 77.8% vs. 13.3%, respectively). These tasks represented the most challenging items of the PBS for the children participating in this study.

The AR-based tasks within the ARMT could be tailored to children’s abilities (e.g., fishing, picking fruit from the tree, labyrinth, and crossing a brook, among others). Furthermore, during the execution of motor tasks in AR, children received brief instructions aimed at promoting an external focus of attention (EFA) facilitated by the applications. Numerous studies have demonstrated the advantages of instructions that induce learners’ EFA, such as focusing on the intended movement effect or outcome, the implement, or the goal of a task, which facilitate motor skill acquisition compared to an internal focus of attention (IFA), which emphasizes concentration on body movements [22,23]. The study by Zorlular et al. [34] reported that EFA-based training was more effective than IFA in improving gait and balance parameters in children aged 6–12 years with unilateral CP. An external focus outside the body can enhance motor automaticity and improve movement effectiveness and efficiency [22,23,35].

Several limitations should be acknowledged. First, the study included a relatively small and clinically heterogeneous sample of children with unilateral and bilateral spastic CP at Gross Motor Function Classification System (GMFCS) levels I–II. Although the sample size was comparable to previous intervention studies in children with CP [5,9,11,21], it limited the precision of the estimates and precluded reliable subgroup analyses. The considerable variability in CP manifestations may have contributed to differential individual responses to rehabilitation [36]. Second, a possible ceiling effect might have an impact on the PBS score, as well as a potential floor effect on the one-legged stance task of the Movement Assessment Battery for Children—Second Edition (MABC-2), which may have reduced their sensitivity to detecting short-term improvements. Additionally, blinding of PBS assessment was not feasible, which could have introduced bias.

Third, five participants from the control group lacked post-intervention data. Consequently, conducting a full intention-to-treat analysis and comparing completers and dropouts was not possible, raising the possibility of attrition bias. Fourth, the ARMT replaced 20–30 min of conventional motor therapy rather than being added to the entire rehabilitation program. In other words, the study aimed to evaluate the effects of combining conventional therapy with AR-based therapy, whereas previous research has primarily examined the effectiveness of VR therapy alone or VR added to conventional training. Therefore, the results reflect the impact of substituting a portion of conventional therapy with ARMT, not its additive effect. Nonetheless, integrating AR-based motor training into standard physiotherapy aligned more closely with typical clinical practices.

Fifth, previous studies involving children with CP have evaluated VR-based training durations ranging from 6 to 12 weeks [37]. Our study sought to determine whether effects could be observed after only 4 weeks of AR-based training. Due to this short duration, larger changes in gait and balance may not have manifested. Lastly, multiple gait and balance outcomes were analyzed without adjustments for multiple comparisons. Consequently, significant findings should be considered exploratory and require confirmation in larger studies with pre-specified primary outcomes.

In summary, this study found no broad additional benefits of replacing part of conventional rehabilitation with four weeks of ARMT in improving gait or balance among children aged 7–12 years with unilateral or bilateral spastic CP at GMFCS levels I–II. The observed effects were limited to specific gait parameters—such as bilateral stride cycle duration, variability in the right first double-support phase, and propulsion index—as well as exploratory indicators of balance improvement. Given the short intervention duration, the combined approach with conventional therapy, and the small sample size, these findings should be interpreted cautiously. Notably, all children assigned to ARMT completed the program, indicating that headset-based AR exercises are feasible in this population. Larger, well-powered trials with pre-specified primary outcomes are necessary to determine whether ARMT can deliver clinically meaningful benefits.

## Figures and Tables

**Table 1:** Basic clinical characteristics of the participants.

Characteristics	ARMT Group (n = 20)	Control Group (n = 15)	Test Statistic and *p*-Value
Age	10.1 ± 2.1 years	9.9 ± 3.0 years	W = 370.5, *p* = 0.739
Sex	Male: 14 (70.0%) Female: 6 (30.0%)	Male: 6 (40.0%) Female: 9 (60.0%)	χ^2^(1) = 3.15, *p* = 0.097
Body height	140.3 ± 15.5 cm	135.5 ± 16.1 cm	W = 390.5, *p* = 0.317
Body weight	33.2 ± 12.2 kg	33.5 ± 11.0 kg	W = 347.0, *p* = 0.677
Unilateral spastic CP	17 (85.0%)	10 (66.7%)	χ^2^(1) = 1.63, *p* = 0.246
Bilateral spastic CP	3 (15.0%)	5 (33.3%)	
GMFCS I	14 (70.0%)	9 (60.0%)	W = 345.0, *p* = 0.557
GMFCS II	6 (30.0%)	6 (40.0%)	
MAS	4.4 ± 4.4 points	4.5 ± 5.1 points	W = 365.0, *p* = 0.878
Gestational age	36.6 ± 6.0 weeks	35.4 ± 5.7 weeks	W = 379.0, *p* = 0.532

Note. Values are mean ± SD or n (%). W = Wilcoxon rank-sum statistic; χ^2^ = Pearson chi-square statistic. *p*-values for χ^2^ tests were estimated using Monte Carlo simulation with 1,000,000 replicates. CP = cerebral palsy; GMFCS = Gross Motor Function Classification System; MAS = Modified Ashworth Scale.

**Table 2:** Descriptive statistics for gait and balance outcomes at pretest and posttest.

Outcome		Unit	Control Pretest		Control Posttest		ARMT Pretest		ARMT Posttest	
			Median	[Q1, Q3]	Median	[Q1, Q3]	Median	[Q1, Q3]	Median	[Q1, Q3]
General spatiotemporal gait parameters										
Cadence	M	steps/min	128.33	[121.46, 139.41]	124.59	[111.77, 133.42]	114.36	[102.78, 122.17]	116.42	[109.09, 123.98]
	CV	%	11.21	[7.50, 16.79]	12.59	[7.49, 15.78]	15.32	[7.75, 17.28]	9.52	[6.91, 14.03]
Speed	M	m/s	1.08	[1.00, 1.24]	1.02	[0.92, 1.21]	0.95	[0.76, 1.17]	1.00	[0.90, 1.10]
	CV	%	1.49	[0.99, 8.20]	2.90	[1.70, 5.71]	1.52	[1.08, 4.70]	1.10	[0.97, 2.17]
Stride length	M	M	1.09	[0.92, 1.14]	1.00	[0.83, 1.23]	1.00	[0.85, 1.15]	1.07	[0.97, 1.12]
	M	% height	73.11	[71.60, 91.06]	74.71	[66.19, 85.95]	72.01	[65.47, 80.14]	75.66	[69.39, 81.60]
	CV	%	7.27	[4.89, 11.75]	7.69	[5.35, 9.58]	7.33	[4.10, 9.51]	5.88	[4.03, 8.11]
Stride cycle duration—left leg	M	S	0.94	[0.88, 1.00]	1.01	[0.90, 1.09]	1.10	[0.99, 1.21]	1.04	[1.00, 1.11]
	CV	%	8.00	[3.91, 10.02]	7.56	[5.47, 9.18]	7.84	[4.83, 9.36]	4.81	[3.58, 6.52]
Stride cycle duration—right leg	M	S	0.94	[0.88, 0.99]	1.01	[0.92, 1.09]	1.11	[0.99, 1.19]	1.05	[0.99, 1.15]
	CV	%	4.26	[3.30, 7.24]	5.31	[3.65, 9.64]	5.74	[3.96, 9.06]	4.88	[3.43, 7.07]
Gait-cycle phase parameters										
Stance phase—left leg	M	% GC	59.90	[58.47, 62.73]	60.55	[60.08, 61.62]	59.73	[56.95, 61.27]	59.71	[57.22, 60.80]
	CV	%	5.41	[2.60, 8.55]	5.67	[3.53, 9.89]	6.98	[5.41, 9.19]	5.16	[3.31, 9.16]
Stance phase—right leg	M	% GC	60.35	[59.30, 61.57]	60.64	[57.98, 61.38]	59.17	[57.45, 61.03]	59.32	[58.22, 61.49]
	CV	%	4.69	[3.74, 9.83]	5.55	[3.40, 7.94]	6.41	[5.28, 9.41]	6.07	[4.51, 7.31]
1st double-support phase—left leg	M	% GC	9.73	[8.30, 11.24]	9.81	[8.63, 11.34]	8.69	[7.31, 10.30]	8.88	[7.89, 11.55]
	CV	%	25.24	[21.64, 27.86]	25.56	[13.00, 37.07]	32.31	[26.65, 35.70]	29.34	[24.97, 39.86]
1st double-support phase—right leg	M	% GC	10.16	[9.73, 12.73]	9.98	[9.03, 12.22]	9.26	[7.71, 10.59]	9.47	[8.30, 10.50]
	CV	%	20.95	[15.58, 28.92]	21.83	[15.90, 25.24]	32.69	[25.98, 46.76]	26.30	[19.84, 33.58]
Single-support phase—left leg	M	% GC	39.31	[37.88, 40.80]	40.78	[38.80, 42.08]	40.70	[39.71, 42.69]	40.55	[39.00, 42.56]
	CV	%	8.83	[6.74, 13.33]	9.01	[7.02, 11.65]	9.02	[7.27, 14.19]	9.69	[7.62, 12.70]
Single-support phase—right leg	M	% GC	40.61	[37.39, 41.86]	39.38	[37.98, 40.40]	40.39	[39.25, 43.86]	40.42	[39.05, 43.13]
	CV	%	11.01	[5.94, 17.94]	12.25	[7.88, 16.82]	12.68	[8.85, 14.39]	8.52	[6.04, 14.37]
Propulsion parameters										
Propulsion index—left side	M	m/s^2^	13.27	[10.55, 14.34]	9.71	[7.62, 12.95]	7.84	[6.96, 9.80]	9.50	[7.60, 10.84]
	CV	%	18.25	[12.07, 26.12]	16.81	[13.50, 28.01]	23.91	[16.00, 41.42]	21.43	[13.84, 30.13]
Propulsion index—right side	M	m/s^2^	11.91	[8.77, 12.81]	8.61	[8.16, 12.84]	7.57	[6.62, 9.10]	8.73	[7.32, 9.33]
	CV	%	27.71	[21.04, 35.37]	23.02	[21.07, 27.17]	28.29	[15.97, 38.81]	22.14	[14.75, 29.36]
Gait quality, pelvic movements, balance										
Walk-quality index—left	M	%	94.87	[94.00, 97.61]	97.98	[94.31, 98.98]	95.17	[92.23, 97.92]	96.07	[93.18, 98.66]
Walk-quality index—right	M	%	97.94	[93.78, 99.19]	96.66	[90.40, 98.46]	96.37	[92.55, 98.26]	96.95	[93.19, 98.57]
Symmetry index	M	%	90.04	[86.22, 93.75]	88.81	[87.48, 92.70]	91.24	[84.30, 93.77]	89.47	[81.08, 95.83]
Tilt symmetry index	M	%	54.10	[29.20, 63.35]	57.60	[30.95, 68.85]	53.40	[40.25, 64.70]	51.40	[41.30, 73.57]
Obliquity symmetry index	M	%	92.70	[88.20, 96.40]	95.60	[83.10, 98.10]	95.20	[91.23, 97.12]	95.50	[92.42, 97.80]
Rotation symmetry index	M	%	95.40	[92.35, 97.55]	94.70	[89.45, 97.75]	95.05	[93.52, 97.10]	95.10	[93.32, 97.20]
Pediatric Balance Scale	M	points	53.00	[48.50, 54.50]	54.00	[51.00, 55.00]	53.00	[52.00, 55.25]	55.00	[53.00, 56.00]
MABC Balance R	M	S	7.00	[4.50, 12.50]	6.00	[4.00, 15.00]	8.00	[5.50, 22.25]	12.00	[6.75, 30.25]
MABC Balance L	M	S	9.00	[6.00, 21.00]	10.00	[5.00, 18.50]	11.50	[3.75, 26.25]	20.00	[8.50, 32.25]

Note. Values are raw, unadjusted participant-level medians, with interquartile ranges reported as [Q1, Q3]. M denotes the participant-level mean or level, and CV denotes the participant-level coefficient of variation. % GC = percentage of the gait cycle.

**Table 3:** Model-estimated changes and between-group effects for gait and balance outcomes.

Outcome		Unit	Control Group			ARMT Group			Diff. in Changes	
			Pretest	Posttest	Change [95% CI]	Pretest	Posttest	Change [95% CI]	[95% CI]	*p*
General spatiotemporal gait parameters										
Cadence	M	steps/min	129.15	125.09	−4.06 [−10.47. 2.34]	113.32	115.74	2.42 [−3.13. 7.97]	6.48 [−1.99. 14.96]	0.129
	CV	%	11.06	11.36	0.30 [−2.55. 3.15]	13.02	10.37	−2.64 [−5.27. −0.01]	−2.94 [−6.82. 0.94]	0.133
Speed	M	m/s	1.14	1.06	−0.08 [−0.24. 0.08]	0.94	0.99	0.05 [−0.07. 0.17]	0.13 [−0.07. 0.34]	0.192
	CV	%	5.18	5.75	0.57 [−3.74. 4.88]	4.46	2.74	−1.73 [−4.25. 0.79]	−2.30 [−7.29. 2.69]	0.356
Stride length	M	M	1.09	1.07	−0.02 [−0.15. 0.11]	1.03	1.05	0.02 [−0.10. 0.13]	0.03 [−0.14. 0.21]	0.694
	M	% height	82.09	80.67	−1.42 [−10.81. 7.97]	73.16	74.93	1.77 [−6.37. 9.90]	3.19 [−9.24. 15.61]	0.605
	CV	%	9.91	8.77	−1.13 [−5.41. 3.15]	8.88	7.01	−1.88 [−5.05. 1.30]	−0.75 [−6.08. 4.59]	0.778
Stride cycle duration—left leg	M	S	0.96	0.99	0.04 [−0.02. 0.10]	1.11	1.06	−0.05 [−0.11. 0.01]	−0.08 [−0.17. 0.00]	0.049
	CV	%	7.53	7.41	−0.12 [−3.08. 2.83]	8.74	5.33	−3.41 [−5.95. −0.86]	−3.28 [−7.18. 0.62]	0.096
Stride cycle duration—right leg	M	S	0.95	1.00	0.05 [−0.02. 0.11]	1.11	1.06	−0.05 [−0.11. 0.01]	−0.10 [−0.19. −0.01]	0.038
	CV	%	5.81	7.20	1.38 [−1.85. 4.62]	7.89	6.26	−1.62 [−4.68. 1.43]	−3.01 [−7.46. 1.44]	0.178
Gait-cycle phase parameters										
Stance phase—left leg	M	% GC	60.48	61.06	0.58 [−1.13. 2.28]	59.24	58.88	−0.36 [−1.84. 1.11]	−0.94 [−3.20. 1.32]	0.403
	CV	%	5.99	6.90	0.91 [−1.32. 3.15]	7.56	5.98	−1.57 [−3.51. 0.37]	−2.49 [−5.45. 0.47]	0.097
Stance phase—right leg	M	% GC	60.69	59.89	−0.80 [−2.77. 1.18]	58.90	60.01	1.12 [−0.59. 2.83]	1.91 [−0.70. 4.53]	0.146
	CV	%	6.72	6.38	−0.34 [−2.20. 1.52]	7.18	6.21	−0.97 [−2.58. 0.64]	−0.63 [−3.09. 1.83]	0.606
1st double-support phase—left leg	M	% GC	9.98	10.37	0.39 [−0.81. 1.59]	8.85	9.55	0.70 [−0.34. 1.74]	0.30 [−1.29. 1.89]	0.701
	CV	%	24.09	27.24	3.14 [−3.35. 9.64]	30.90	30.71	−0.20 [−5.82. 5.43]	−3.34 [−11.93. 5.26]	0.435
1st double-support phase—right leg	M	% GC	11.02	10.48	−0.54 [−1.94. 0.86]	8.97	9.31	0.34 [−0.87. 1.56]	0.89 [−0.97. 2.74]	0.338
	CV	%	22.67	24.16	1.49 [−4.92. 7.90]	34.26	26.84	−7.42 [−12.97. −1.87]	−8.91 [−17.39. −0.44]	0.040
Single-support phase—left leg	M	% GC	39.33	40.28	0.95 [−1.04. 2.94]	41.57	40.15	−1.42 [−3.15. 0.31]	−2.37 [−5.01. 0.27]	0.077
	CV	%	9.42	9.53	0.12 [−1.93. 2.16]	10.48	10.24	−0.23 [−2.17. 1.70]	−0.35 [−3.16. 2.46]	0.801
Single-support phase—right leg	M	% GC	39.70	39.15	−0.55 [−2.31. 1.21]	41.01	41.01	−0.01 [−1.53. 1.52]	0.54 [−1.79. 2.88]	0.639
	CV	%	10.86	11.63	0.77 [−2.72. 4.26]	12.45	9.67	−2.78 [−5.80. 0.24]	−3.56 [−8.17. 1.06]	0.127
Propulsion parameters										
Propulsion index—left side	M	m/s^2^	12.00	10.46	−1.54 [−3.24. 0.16]	8.09	8.97	0.88 [−0.23. 2.00]	2.42 [0.39. 4.45]	0.021
	CV	%	19.69	20.80	1.11 [−5.31. 7.53]	27.60	22.14	−5.46 [−12.37. 1.46]	−6.57 [−16.00. 2.86]	0.166
Propulsion index—right side	M	m/s^2^	11.17	10.53	−0.64 [−1.92. 0.64]	7.73	8.47	0.74 [−0.09. 1.57]	1.38 [−0.14. 2.91]	0.074
	CV	%	27.74	23.86	−3.89 [−12.03. 4.25]	28.12	22.76	−5.35 [−12.35. 1.64]	−1.47 [−12.20. 9.27]	0.783
Gait quality, pelvic movements, balance										
Walk-quality index—left side	M	%	93.26	95.05	1.79 [−3.25. 6.83]	92.29	94.75	2.45 [−2.41. 7.32]	0.66 [−6.34. 7.67]	0.849
Walk-quality index—right side	M	%	96.53	94.35	−2.18 [−5.68. 1.32]	94.33	94.68	0.35 [−3.20. 3.90]	2.53 [−2.46. 7.52]	0.310
Symmetry index	M	%	89.66	89.29	−0.37 [−4.50. 3.77]	89.52	89.99	0.47 [−3.02. 3.96]	0.83 [−4.58. 6.25]	0.756
Tilt symmetry index	M	%	49.61	52.08	2.47 [−8.71. 13.66]	51.92	56.27	4.35 [−5.34. 14.04]	1.88 [−12.92. 16.67]	0.798
Obliquity symmetry index	M	%	92.44	92.77	0.33 [−3.11. 3.76]	93.56	94.80	1.25 [−1.14. 3.63]	0.92 [−3.26. 5.10]	0.657
Rotation symmetry index	M	%	93.88	94.34	0.47 [−1.83. 2.77]	94.66	94.88	0.22 [−1.54. 1.99]	−0.24 [−3.14. 2.66]	0.865
Pediatric Balance Scale	M	Points	53.19	53.50	0.31 [−0.65. 1.27]	54.09	54.81	0.72 [−0.01. 1.45]	0.41 [−0.79. 1.61]	0.502
MABC Balance R	M	S	8.69	7.12	−1.57 [−4.91. 1.77]	8.62	12.01	3.39 [−0.65. 7.44]	4.96 [−0.28. 10.21]	0.063
MABC Balance L	M	S	11.48	11.47	−0.01 [−4.85. 4.84]	11.79	18.66	6.88 [0.57. 13.19]	6.89 [−1.06. 14.83]	0.088

Note. Pretest and posttest values are model-estimated values back-transformed to the original response scale. Change = posttest − pretest. Difference in changes = (ARMT posttest − ARMT pretest) − (control posttest − control pretest). Confidence intervals and two-sided, unadjusted *p*-values were obtained for these contrasts on the original response scale using a delta-method Wald approximation. Positive values indicate a relatively greater increase in ARMT; whether an increase or decrease is favorable depends on the outcome. M denotes the participant-level mean or level, and CV denotes the participant-level coefficient of variation. % GC = percentage of the gait cycle.

## Data Availability

Data are available on request due to privacy and ethical restrictions. The data presented in this study are available on request from the corresponding author.

## Abbreviations

The following abbreviations are used in this manuscript:

apps	Applications
AR	Augmented reality
ARMT	Augmented reality motor training
CP	Cerebral palsy
EFA	External focus of attention
HMD	Head-mounted device
IFA	Internal focus of attention
MABC-2	Movement Assessment Battery for Children, 2nd edition Test
PBS	Pediatric Balance Scale
SW	Software
VR	Virtual reality
